# Enhanced Thermal Insulation of the Hollow Glass Microsphere/Glass Fiber Fabric Textile Composite Material

**DOI:** 10.3390/polym13040505

**Published:** 2021-02-07

**Authors:** Jintao Sun, Fei Cai, Dongzhi Tao, Qingqing Ni, Yaqin Fu

**Affiliations:** 1 Key Laboratory of Advanced Textile Materials and Manufacturing Technology, Ministry of Education, Zhejiang Sci-Tech University, Hangzhou 310018, China; sjtt1015@163.com (J.S.); caifei_PG@126.com (F.C.); itisamber@163.com (D.T.); 2 Department of Mechanical Engineering & Robotics, Shinshu University, Ueda 386-8567, Japan; niqq@shinshu-u.ac.jp

**Keywords:** hollow glass microsphere (HGM), thermal insulation, glass fiber fabric, composites

## Abstract

Glass fiber fabrics/hollow glass microspheres (HGM)–waterborne polyurethane (WPU) textile composites were prepared using glass fiber, WPU, and HGM as skeleton material, binder, and insulation filler, respectively, to study the effect of HGM on the thermal insulation performance of glass fiber fabrics. Scanning electron microscopy, Instron 3367 tensile test instrument, thermal constant analysis, and infrared thermal imaging were used to determine the cross-sectional morphology, mechanical property, thermal conductivity, and thermal insulation property, respectively, of the developed materials. The results show that the addition of HGM mixed in WPU significantly enhanced thermal insulation performance of the textile composite with the reduction of thermal conductivity of 45.2% when the volume ratio of HGM to WPU is 0.8 compared with that of material without HGM. The composite can achieve the thermal insulation effect with a temperature difference of 17.74 °C at the temperature field of 70 °C. Meanwhile, the tensile strength of the composite is improved from 14.16 to 22.14 MPa. With these results, it is confirmed that designing hollow glass microspheres (HGM) is an effective way to develop and enhance the high performance of insulation materials with an obvious lightweight of the bulk density reaching about 50%.

## 1. Introduction

Global warming has become a hot issue since its first proposal in the 1970s. Excessive human activities, such as the use of fossil fuels, are one of the main causes [1,2,3,4,5]. Due to the limited energy sources and environmental degradation, energy saving has become compulsory [6]. Energy management through the use of thermal insulation materials, which is an important method in alleviating the global warming trend, can effectively reduce energy consumption [7]. Some organic insulation materials, such as expanded polystyrene [8,9,10] and rigid polyurethane [11,12,13,14] foams, are flammable and fire hazards, thereby limiting their application. By contrast, the use of inorganic thermal insulation materials has been increasing. The thermal conductivity of composite materials is usually reduced by adding thermal insulation fillers to achieve thermal insulation. The thermal conductivity of air, which is one of the materials with the lowest thermal conductivity in nature, is about 0.023 W/(m·K) [15]. Therefore, the hollow structure of the fillers as an effective way is used for storing a large amount of air to facilitate the preparation of barrier thermal insulation materials.

As a kind of high-temperature-resistant inorganic fibrous thermal insulation material, glass fiber fabric is widely used in the chemical industry, construction, fire protection, and other industrial fields [16]. However, as a traditional thermal insulation material, glass fiber fabric has relatively high thermal conductivity, high density, and general thermal insulation performance [17]. Therefore, the enhancement of its thermal insulation performance and the reduction in its overall density for lightweight in reducing energy consumption have become a key issue that needs to be resolved. Hollow glass microspheres (HGM), which are spherical and lightweight inorganic capsule, are widely used in some fields, such as coatings [18], plastics [19], aerospace [20,21], hydrogen storage [22,23], and building materials [24]. Having the advantages of isotropy, low density, good chemical stability, good moisture resistance, small and well-distributed internal stress, and low thermal conductivity, HGM have become a lightweight thermal insulation filler with a wide range of use and excellent performance [25,26,27,28,29,30,31,32]. The positive impacts of HGM addition on lowering the thermal conductivity and flammability of composites have been demonstrated [33,34,35]. The tiny spherical structure of HGM has the advantages of better fluidity, dispersibility, and isotropy, than flake-, needle-, or irregular-shaped fillers. The addition of HGM as a filler can effectively control the shrinkage of the product and dimensionally stabilize the product in all directions without warping. Combining HGM as a filler and a matrix material reduces the weight of the composite material and improves the thermal insulation performance [36]. Researchers have done a lot of work in related research by using HGM as fillers in combination with polypropylene, phenolic resin, epoxy resin, silicone rubber, high-density polyethylene, and other organic matrices to study the thermal insulation effect [37,38,39,40,41,42]. Besides, thermal insulation materials are generally able to withstand various physical and chemical changes and mechanical effects at high temperatures. Therefore, good mechanical properties are necessary for the use of thermal insulation composites.

In this study, hollow glass microspheres are added to glass fiber fabric to incorporate the superior features of HGM such as low density and low thermal conductivity in conventional textile products to achieve the preparation of lightweight thermal insulation textile composite materials. A series of textile composites with different HGM/waterborne polyurethane(WPU) volume ratios were prepared, and the mechanical properties and thermal insulation properties were evaluated.

## 2. Materials and Methods

### 2.1. Materials

HGM were purchased from Minnesota Mining and Manufacturing, St. Paul, MN, USA. The physical properties of HGM are shown in Table 1. Glass fiber fabrics (*ρ* = 1.1 g/cm^3^) and WPU (solid content = 46%) were purchased commercially. Ethanol was purchased from Aladdin Industrial Corporation, Shanghai, China. All chemicals were used as received without further purification.

### 2.2. Methods

**Figure 1:**
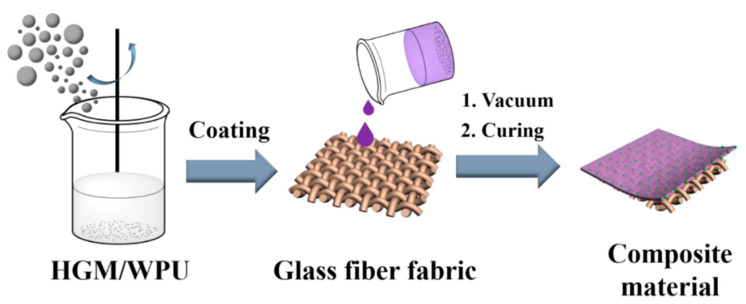
Preparation of glass fiber fabric/ hollow glass microspheres–waterborne polyurethane thermal insulation textile composites.

The masses of HGM required were calculated by different ratios. After washing thrice with ethanol, HGM were dried in a 60 °C blast-drying oven. Figure 1 shows the preparation of the textile composite. HGM and WPU were mixed, and the mixture was stirred slowly for a while. The ratio of HGM to WPU was referred to as the volume ratio. The mixture was evenly coated on the surface of glass fiber fabrics with a thickness of 0.7 mm through the coating method. The vacuum drying box was used to remove the bubbles from the textile composite. The textile composite was placed in an oven maintained at 60 °C for 4–6 h and cooled to room temperature. Afterward, the textile composite could be processed into the required sizes, and the corresponding structural performance was observed. The thickness of the final textile composite was controlled at 2 mm. The volume content of the glass fiber fabric in the textile composite was 35%.

### 2.3. Characterization

The surface morphologies of HGM and the manufactured samples were observed using field-emission scanning electron microscopy (FESEM, ZEISS VLTRA-55, Jena, Germany). The particle size of HGM was measured using a laser particle size analyzer (Mastersizer 2000, Malvern City, United Kingdom). The viscosity of the HGM–WPU mixture with different proportions was analyzed using a rotary rheometer (Thermo Fisher Scientific, Waltham, MA, USA). The thermal conductivity of the textile composite was measured using a thermal constant analyzer (TPS 2500S, Shanghai K-Analysis Co., Ltd., Shanghai, China). The drainage method was used to measure the bulk density of the textile composite. The tensile strength was investigated by a universal tensile testing machine (Instron-3367, Instron, Norwood, MA, USA) at room temperature, with a cross-head speed of 20 mm/min. The infrared thermal imaging (InfraTec, Dresden, Germany) was used to test the thermal insulation performance of the textile composite. The working distance was 40 cm. The temperature of the sample was detected and recorded using a thermocouple connected to a temperature controller.

## 3. Results and Discussion

**Figure 2:**
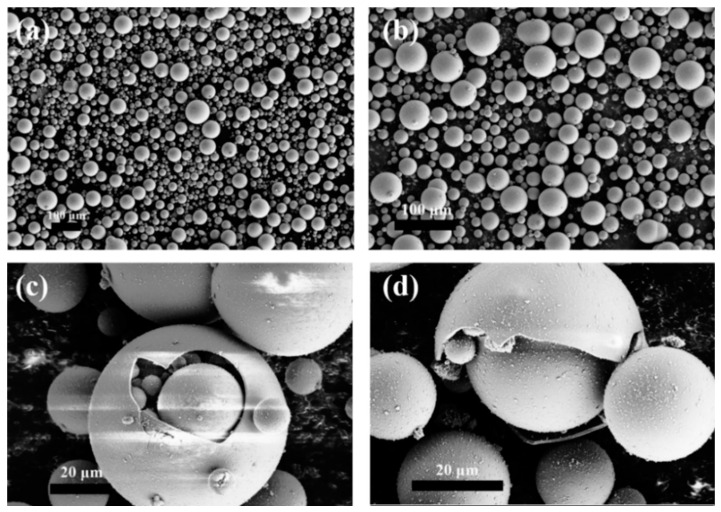
FESEM images of hollow glass microspheres at the resolution of (**a**) 100×, (**b**) 200×, and (**c**,**d**) 1000×.

**Figure 3:**
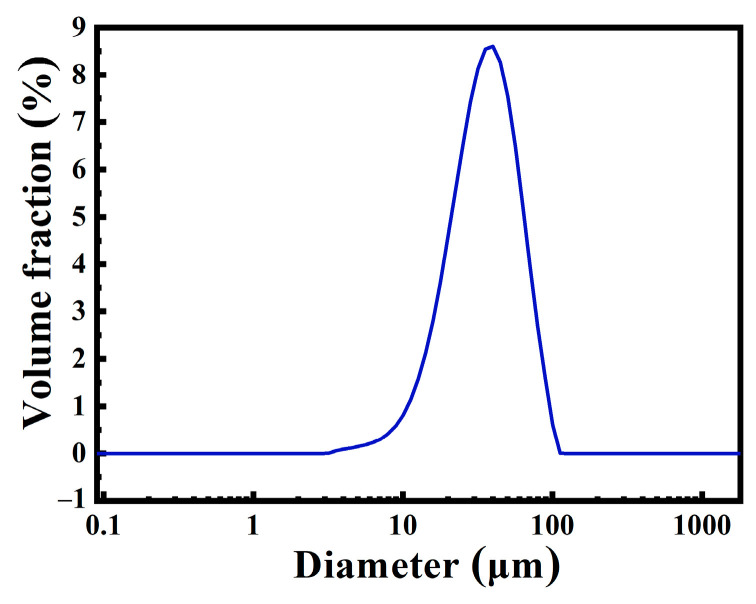
The particle size distribution of hollow glass microspheres.

The diameter of the commercially purchased HGM was not uniform, and the particle size distribution was relatively wide, as shown in Figure 2a,b. A laser particle size analyzer was used to determine the particle size distribution of HGM. Figure 3 shows that the particle size distribution of VS5500 HGM was 10–100 μm, which was approximately normal. Figure 2c shows that the cavity of HGM contained air and some smaller HGM with multicapsules. This special structure allowed the external heat to be reflected multiple times inside the HGM, and part of the heat entered the smaller capsule in the HGM, which further reduced the heat transfer efficiency and enhanced the thermal insulation performance of the textile composite. The damage in Figure 2d shows that the HGM had a thin wall and a large cavity volume, which had great advantages as a barrier type thermal insulation filler.

**Figure 4:**
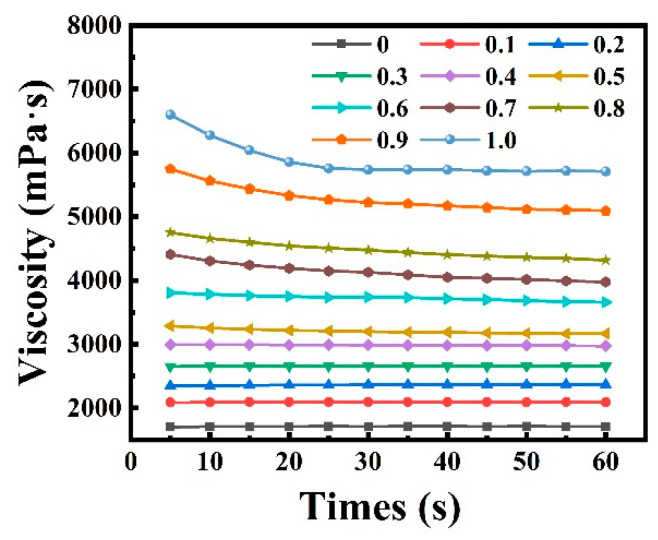
The viscosity of the mixture with different volume ratios of hollow glass microspheres and waterborne polyurethane.

The viscosity of the HGM–WPU mixture with different ratios was determined at 20 °C Figure 4 shows the relationship between the viscosity and the rotation time of the mixture at different formulations. As the volume fraction of HGM in the mixture increased, the viscosity of the HGM–WPU mixture gradually increased. This finding also indicates that the fluidity of the mixture gradually decreased, which was not conducive to the processing of composite materials. The same conclusion can be obtained during the sample preparation process. With increasing volume fraction of HGM, stirring the mixture became difficult. Moreover, the coating became difficult, thereby affecting the final molding effect of the composite material. The WPU had an average viscosity of 1711 mPa·s and certain fluidity. When the volume ratio of HGM/WPU was 1, the average viscosity of the mixture reached 5845 mPa·s, resulting in difficulty in preparing composite materials by using the coating method.

**Figure 5:**
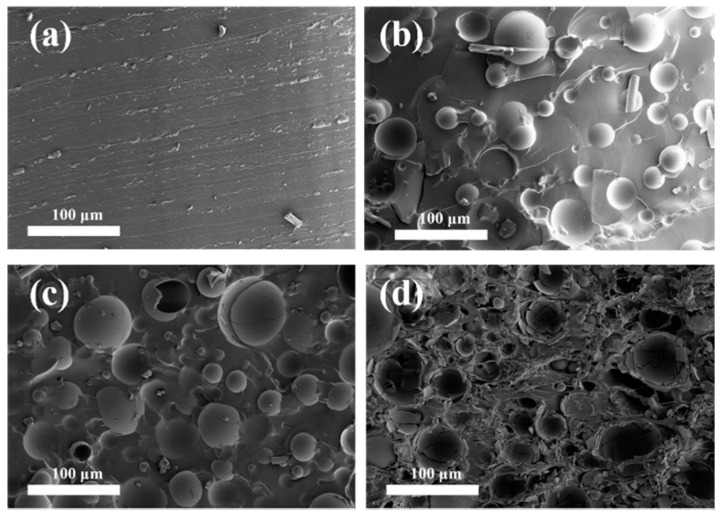
Cross-section FESEM images of composites with hollow glass microsphere/waterborne polyurethane volume ratios of (**a**) 0, (**b**) 0.1, (**c**) 0.4, and (**d**) 0.7.

**Figure 6:**
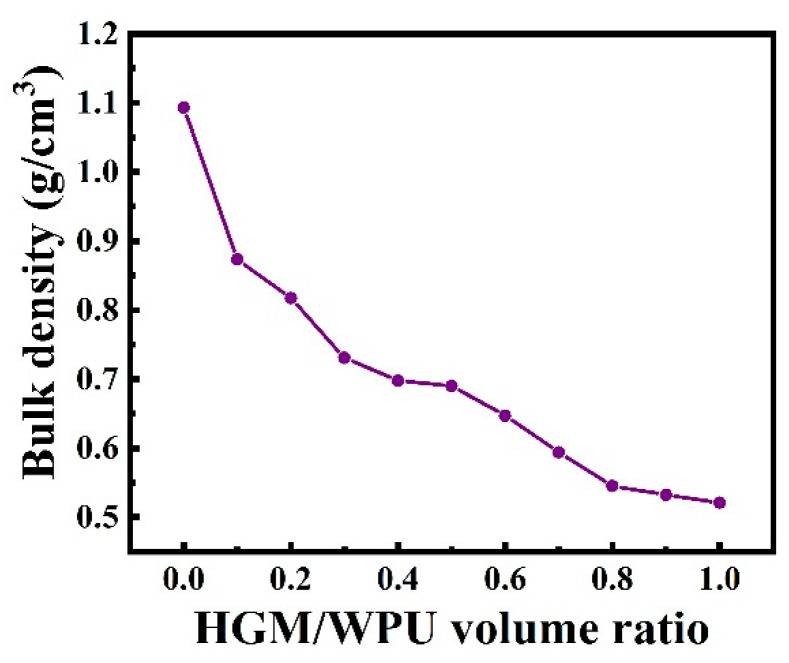
Effects of the hollow glass microspheres volume fraction on the bulk density of the textile composite.

Commercial HGM are generally low in mechanical property and prone to cracking under pressure. After mixing HGM with the resin, the flexibility of the resin can be used to relieve the cracks caused by pressure to a certain extent. As a result, WPU acted as a binder and protected HGM. The cross-sectional morphology of the textile composite is shown in Figure 5. When the ratio of HGM/WPU was only 0.1, HGM were completely wrapped in WPU and evenly dispersed throughout the resin matrix [43] (Figure 5b). As such, HGM did not break during the preparation of the cross-section sample for FESEM. As the volume fraction of HGM increased, the probability of contact increased. Collision was easy and caused HGM to rupture, thereby affecting the thermal insulation effect in the textile composite. Insufficient resin was present for protection due to the excessive number of HGM, resulting in the rupture of the majority of HGM (Figure 5d). Figure 6 shows the bulk density of textile composites with different ratios. When the ratio reached 0.8, the volume density of the textile composite (0.54 g/cm^3^) was reduced by 50.9% compared with that of material without HGM (1.1 g/cm^3^). The density of HGM was smaller than that of other components in the composite. With an increased volume fraction of HGM, the content of other components decreased under a certain thickness, decreasing the overall density of the composite, which is advantageous for the preparation of lightweight insulation materials. Moreover, during the preparation of the textile composite, the mechanical mixing of the HGM and WPU generated a small number of bubbles. Although a vacuum drying box was used to remove some bubbles during the coating molding process, some bubbles remain, thereby decreasing the bulk density of the composite material.

**Figure 7:**
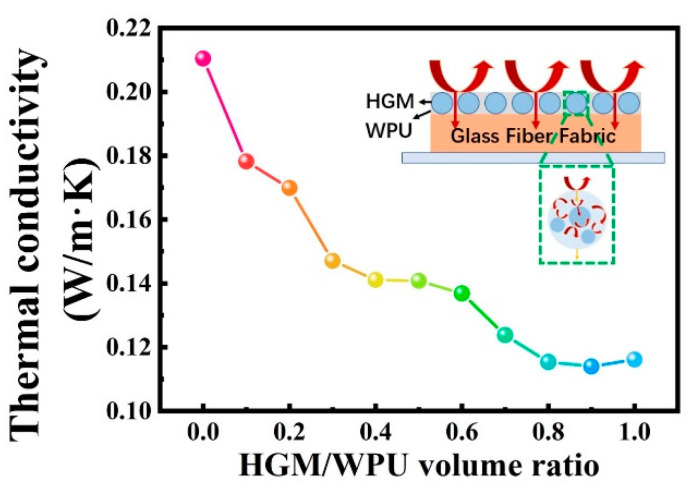
Effects of hollow glass microspheres volume fraction on the thermal conductivity. The insert image is a thermal insulation mechanism diagram of the textile composite.

Figure 7 shows the effect of the different volume ratios of HGM/WPU on the thermal conductivity of the material. As the volume fraction of HGM increased, the thermal conductivity of the textile composite decreased. At volume ratios of 0.4 and 0.8, the thermal conductivity values of the textile composite were 0.1411 and 0.1154 W/(m·K), respectively, thereby showing a reduction of 32.9% and 45.2%, respectively, compared with that of the material without HGM (0.2104 W/(m·K)). These results indicate that the addition of HGM affected the improvement of thermal insulation performance. However, when the ratio exceeded 0.8, increasing the content did not result in a considerable decrease in the thermal conductivity of the textile composite. Solid heat conduction, heat radiation between adjacent hollow microspheres, and heat convection inside the hollow microspheres occur in the prepared textile composite. When the content of HGM reaches the limit, the three main heat transfer paths in the textile composite no longer changes, and the overall insulation performance of the material remains unchanged [44,45].

**Figure 8:**
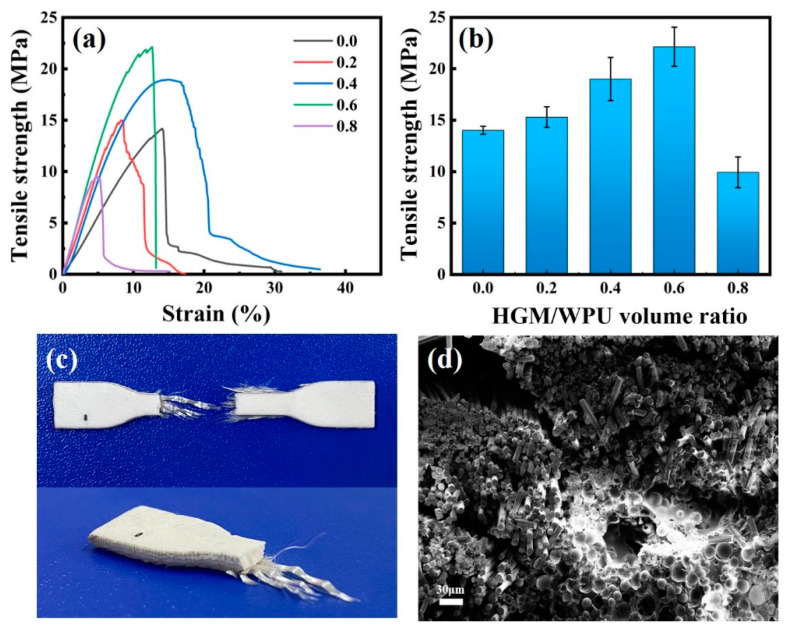
(**a**) Typical stress-strain curves and (**b**) tensile strength of textile composites with different hollow glass microspheres/waterborne polyurethane volume ratios. (**c**) Digital photo and (**d**) SEM image of the cross-section after a tensile fracture.

Thermal insulation composite materials must not only have excellent thermal insulation properties but also have good mechanical properties. The textile composites of the different volume ratios (0–0.8) and the same size have been prepared to investigate the effect of the volume ratios of HGM/WPU on the mechanical properties. A dumbbell-shaped cutter was used to cut the textile composite into a dumbbell-shaped specimen (25 mm × 4 mm × 2 mm), and the mechanical properties were tested using a universal tensile testing machine (Instron-3367, Instron, Norwood, MA, USA) under a tensile speed of 20 mm/min at room temperature. At least five effective specimens were tested for each sample. As seen from Figure 8, as the volume ratio increased, the tensile strength of the textile composite gradually increased. When the volume ratio reached 0.6, the best tensile strength was 22.14 MPa, which was 56.3% higher than that of material without HGM. The tensile strength of composite materials depended on the combined effect of WPU and HGM. When the volume ratio was 0, the main role was mainly the WPU matrix. As the amount of HGM continued to increase, the role of HGM continued to highlight. From the stress-strain diagram, it could be concluded that when the volume ratio was 0.6, the bonding between the water-based polyurethane mixed with hollow glass microspheres and the glass fiber fabric achieved a good effect. As the volume ratio continued to increase to 0.8, the tensile strength was greatly reduced, even lower than the glass fiber fabric with only WPU added (14.16 MPa). When the amount of HGM added reached a certain value, the combination of WPU and HGM reached saturation. Moreover, when the volume ratio reached 0.8, stirring of the mixture became particularly difficult, more bubbles were generated, and the coating effect was poor. Therefore, the tensile strength of the textile composite materials had a downward trend.

**Figure 9:**
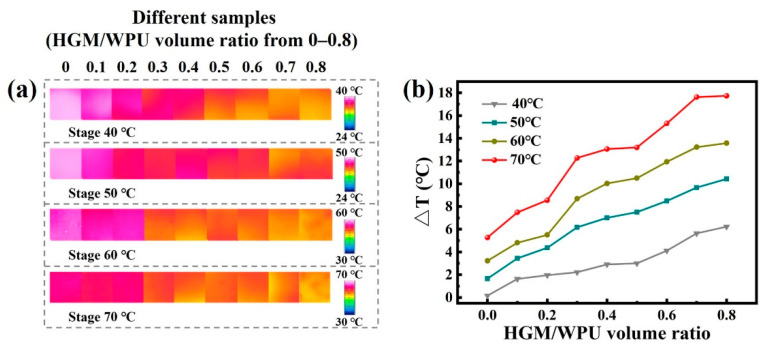
(**a**) Infrared thermal images of textile composites with different hollow glass microspheres/waterborne polyurethane volume ratios. (**b**) The thermal insulation performance of composites with different hollow glass microspheres/waterborne polyurethane volume ratios at different temperatures.

At volume ratios of 0.9 and 1.0, the thermal conductivity of the textile composite remained stable, and the thermal insulation performance did not change remarkably. As a result, only samples with a volume ratio of 0–0.8 were selected for the thermal insulation performance test. At ambient temperature (23 °C) and relative humidity of 50%, the textile composite was placed on a constant-temperature heating table. When the temperature of the sample surface became stable after a few minutes, the images were obtained under the infrared thermal imaging system and are presented in Figure 9a. As summarized in Figure 9b, the temperature difference between the surface of the same composite material sample and the heating stage gradually increased with increasing test temperature. At a temperature field of 70 °C and a ratio of 0.8, the composite material achieved a temperature difference of 17.74 °C, which was improved by 235% compared with that of the material without HGM. No evident artificial bubble was observed in the prepared composite material. When the bubble diameter was less than 4 mm, no natural convection was observed in the bubble. Thus, the influence of the bubble generated during the preparation process on the heat convection could be ignored. Similarly, the diameter of HGM we selected was basically below 0.1 mm, and the natural convection of gas in the glass microspheres could be ignored. Besides, polymer composites are usually tested at low temperatures, and the proportion of heat radiation in the total heat transfer is extremely small. Therefore, the solid heat transfer has the greatest effect on the thermal insulation performance [46]. At volume ratios of 0.4 and 0.5, the textile composite had densities of 0.697 and 0.690 g/cm^3^, respectively and thermal conductivities of 0.1411 and 0.1408 W/(m·K), respectively. Thus, the thermal conductivity of textile composites was almost the same when the bulk density was similar.

## 4. Conclusions

In summary, HGM were added to the glass fiber fabric through a simple coating process, successfully preparing lightweight thermal insulation textile composite materials. The thermal conductivity of the textile composite was reduced by 45.2% when the volume ratio of HGM to WPU was 0.8. The composite with an obvious lightweight of the bulk density reaching about 50% could achieve the thermal insulation effect with a temperature difference of 17.74 °C at the temperature field of 70 °C. Meanwhile, the tensile strength of the composite was improved from 14.16 to 22.14 MPa. This work may provide new ideas for the preparation of new thermal insulation materials in the future.

## Figures and Tables

**Table 1:** Physical properties of the hollow glass microspheres.

Model	Density (g/cm^3^)	Compressive Strength (MPa)	Thermal Conductivity (W/(m·K))	Color
VS5500	0.38	37.9	0.127	white

## Data Availability

The data presented in this study are available on request from the corresponding author.
